# Epidemiology of Enterovirus Genotypes in Association with Human Diseases

**DOI:** 10.3390/v16071165

**Published:** 2024-07-19

**Authors:** Zhenfeng Xie, Pattara Khamrin, Niwat Maneekarn, Kattareeya Kumthip

**Affiliations:** 1 Department of Microbiology, Faculty of Medicine, Chiang Mai University, Chiang Mai 50200, Thailand; xiezhenfeng22@gmail.com (Z.X.); pattara.k@cmu.ac.th (P.K.); niwat.m@cmu.ac.th (N.M.); 2 Guangxi Colleges and Universities Key Laboratory of Basic Research and Transformation of Cancer Immunity and Infectious Diseases, Youjiang Medical University for Nationalities, Baise 533000, China; 3 Center of Excellence in Emerging and Re-Emerging Diarrheal Viruses, Chiang Mai University, Chiang Mai 50200, Thailand

**Keywords:** epidemiology, enterovirus, genotypes, infectious diseases

## Abstract

Enteroviruses (EVs) are well-known causes of a wide range of infectious diseases in infants and young children, ranging from mild illnesses to severe conditions, depending on the virus genotypes and the host’s immunity. Recent advances in molecular surveillance and genotyping tools have identified over 116 different human EV genotypes from various types of clinical samples. However, the current knowledge about most of these genotypes, except for those of well-known genotypes like EV-A71 and EV-D68, is still limited due to a lack of comprehensive EV surveillance systems. This limited information makes it difficult to understand the true burden of EV-related diseases globally. Furthermore, the specific EV genotype associated with diseases varies according to country, population group, and study period. The same genotype can exhibit different epidemiological features in different areas. By integrating the data from established EV surveillance systems in the USA, Europe, Japan, and China, in combination with other EV infection studies, we can elaborate a better understanding of the distribution of prevalent EV genotypes and the diseases associated with EV. This review analyzed the data from various EV surveillance databases and explored the EV seroprevalence and the association of specific EV genotypes with human diseases.

## 1. Introduction

Enteroviruses (EVs) are a highly contagious group of viruses that cause a wide range of diseases, including hand, foot, and mouth disease (HFMD), encephalitis, aseptic meningitis, myocarditis, conjunctivitis, and respiratory and gastrointestinal tract diseases. While most EV infections cause no symptoms, some infections can cause severe diseases in newborns, young children, and immunocompromised patients [1,2]. The rapid decline of maternal antibody titer after birth makes most neonates a population susceptible to EV infection [3]. Over one hundred different EV genotypes have been identified, and the specific genotype associated with a particular disease can vary significantly based on country, population group, and the period of the study. Certain clinical symptoms linked to specific EV types are well-documented. For example, enterovirus A71 (EV-A71) and coxsackievirus A16 (CVA16) are known to cause HFMD, while EV-D68 is associated with respiratory illnesses and acute flaccid myelitis (AFM) [4,5]. However, the clinical significance of many other EV types remains unclear.

Over the past few decades, the Asia-Pacific region has consistently experienced outbreaks of HFMD, primarily caused by several genotypes of EV species A (EV-A), such as EV-A71, CVA16, CVA6, and CVA10 [6]. Even though Europe has also reported outbreaks of HFMD caused by CVA6 and EV-A71, the scale of the outbreaks appeared much smaller compared to those in the Asia-Pacific region [6]. In the United States, outbreaks of respiratory illnesses caused by EV-D68 have occurred every two years between 2014 and 2018 [7], while HFMD outbreaks are less frequently reported. Due to the lack of a coordinated global surveillance system for non-polio EV, unlike the highly successful Global Polio Laboratory Network (with 146 laboratories in 92 countries), the current understanding of the true impact of EV infections across different regions is limited. This highlights the urgent need for the improvement of EV surveillance covering different geographical regions to gain a clearer picture of the global distribution of EV infections, the disease burden caused by different genotypes, and ultimately the guidelines for vaccine development.

To date, several countries have established dedicated EV surveillance systems. Japan utilizes a sentinel-based system to monitor the cases of aseptic meningitis, HFMD, and herpangina [8]. China focuses mainly on monitoring HFMD cases [9]. The United States relies on a voluntary reporting system for both EV cases and genotyping data [10]. Additionally, Europe has conducted a comprehensive analysis of EV infection cases across 24 countries within Europe and the European Economic Area [11]. Surveillance databases offer several advantages over published studies. They typically collect data from a wider and larger population, providing more specific information on disease prevalence. Additionally, these databases are updated more frequently and strictly to data quality control standards, minimizing the risk of inaccurate or incomplete data. This review aims to describe the distribution of EV infection based on the data from different EV surveillance databases and to explore the EV seroprevalence and the association of specific EV genotypes with human diseases.

## 2. The Classification, Genomic Organization, and Structure of Enteroviruses

Enteroviruses (EVs) are members of the *Enterovirus* genus, belonging to the family *Picornaviridae.* EVs are classified into fifteen species, including twelve species of *Enterovirus* (EV-A to EV-L) and three species of *Rhinovirus* (RV-A to RV-C) [12]. Among these, EV-A to EV-D and RV-A to RV-C infect humans. Within EV-A to EV-D, 116 genotypes have been genetically characterized through phylogenetic analysis of their viral protein 1 (VP1) sequences [13,14]. Specifically, EV-A comprises 25 genotypes, and EV-B, EV-C, and EV-D comprise 63, 23, and 5 genotypes, respectively [12,15].

**Figure 1:**
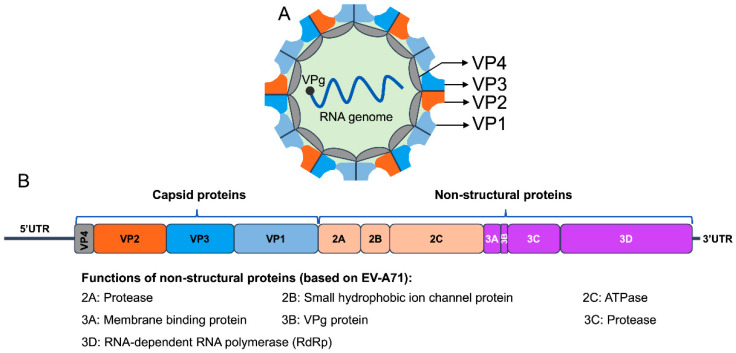
The virion structure of enterovirus (**A**). The genome structure of enterovirus (**B**).

EVs are non-enveloped viruses containing a positive-sense, single-stranded RNA genome with an icosahedral symmetry virion of about 22–30 nm in diameter [16]. The EV capsid comprises four viral proteins (VP1 to VP4) that function as structural components, forming a protective shell surrounding the RNA genome. The viral capsid consists of a densely packed icosahedral arrangement of 60 structural subunits (protomers); each protomer consists of four structural proteins, VP1 to VP4. The VP1, VP2, and VP3 are exposed on the surface of the capsid, while VP4 is located internally and serves as a structural stabilizer [16] (Figure 1A). The surface-exposed VP1-VP3 proteins are crucial for eliciting an immune response, as they contain most neutralizing antigenic epitopes [17,18,19,20].

The RNA genome of EVs is about 7100 to 7450 nucleotides (nt) in length and shares a conserved organization and gene expression strategy within the genus [21,22]. The EV genome contains a single large open reading frame (ORF) flanked by a 5′ untranslated region (5′UTR) and a 3′UTR [23] (Figure 1B). The ORF encodes a single viral polyprotein, which is initially processed by viral proteases into three precursor proteins, P1, P2, and P3. The P1 precursor is proteolytically cleaved into the structural capsid proteins (VP1–VP4). The P2 and P3 are processed into nonstructural proteins that are required for protein processing and genome replication, including the proteases 2A, 3C, and 3CD, the RNA-dependent RNA polymerase 3D, and the RNA synthesis primer 3B (VPg) [24,25]. The organization of the EV polyprotein has a consistent pattern, beginning with the capsid proteins (VP4, VP2, VP3, and VP1) at the amino terminus of the polypeptide, followed by the nonstructural proteins (2A, 2B, 2C, 3A, 3B, 3C, and 3D) [23]. In poliovirus and EV-A71, the 3′UTR contains stem-loop structures that regulate the replication of the virus and end with a poly-A tail of variable length [26,27,28].

## 3. Transmission and Clinical Features

EVs are highly contagious and primarily spread through the fecal-oral route [29]. Other transmission routes include respiratory secretions and direct contact with contaminated objects before touching the mouth, nose, or eyes [30,31]. However, some acid-sensitive EVs, such as EV-D and RV-A to RV-C, are most likely to be transmitted via the respiratory route [32,33]. EV infections can present a wide range of clinical manifestations, from asymptomatic to severe conditions, depending on the virus genotype and the host’s immune system [34]. The majority of EV infections are asymptomatic; however, younger children commonly experience mild illnesses, such as respiratory disease, conjunctivitis, herpangina, hand, foot, and mouth disease, pleurodynia, fever, and rash [34]. Additionally, EVs can cause serious complications, especially in infants, younger children, and immunocompromised individuals [34,35]. Severe complications include acute flaccid paralysis, meningitis, encephalitis, myocarditis, and neonatal sepsis [36,37]. The specific tissues and organs affected by EV infections are dependent on the genotype of the virus. They can target the central nervous system (CNS), skin, eyes, pharynx, heart, and respiratory and gastrointestinal tracts [31,36].

## 4. Distribution of Enterovirus Infections

Data summarized from enterovirus surveillance systems in the USA, Japan, China, and 24 European countries are shown in Table 1. Overall trends of enterovirus infections reported by major surveillance systems in the past decade in different regions demonstrated the co-circulation of EV-A, EV-B, and EV-D in the USA, Japan, and Europe. In contrast, the enterovirus surveillance system in China primarily monitors hand, foot, and mouth disease (HFMD) cases, which are predominantly caused by EV-A, and lacks comprehensive data on other EV species. However, the distribution and prevalence of EV species and genotypes vary depending on the country and the study period. Across different regions, the most common EV genotypes reported were CVA6, CVA16, EV-A71, and EV-D68. EV-A (notably CVA6 and EV-71) is frequently reported, particularly in association with HFMD. EV-D68 is remarkably prevalent in the USA, primarily linked with respiratory illnesses and acute flaccid myelitis.

### 4.1. Enterovirus Infections in the USA

EVs, including EV-A, EV-B, and EV-D, are commonly found throughout the USA [38,39]. From 2014 to 2016, CVA6, E30, E18, CVB3, and EV-D68 were the five most frequently detected genotypes [38]. Notably, EV-D68 caused significant outbreaks in 2014 and 2016, resulting in 1542 cases of severe respiratory illness and 277 cases of acute flaccid myelitis (AFM) in children [5,38,40]. During this period, EV-D68 infection was the main burden of EV infections (55.9%), followed by infections caused by several EV-B genotypes (E30, E18, and CVB3) at 14.6%. CVA6, predominantly associated with HFMD, accounted for 6.1% of the total reported cases. The most common type of specimen reported was from a throat or nasopharyngeal swab (70.0%).

**Table 1:** Enterovirus infections reported by major surveillance systems between 2013 and 2023.

Surveillance System: Country	Period [Refs.]	EV Species	Most Prevalent Genotypes	Proportion	Reported Diseases (Proportion, No. of Cases)	Specimen Type
National Enterovirus Surveillance System (NESS): USA	2014–2016 [5,38,40]	EV-A	CVA6	6.1%	Hand-foot-mouth disease *	CSF: 18.8% T/NS: 70.0%
		EV-B	CVB3, E18, E30	14.6%	NA	
		EV-D	EV-D68	55.9%	Respiratory illnesses (1542 cases) Acute flaccid myelitis (276 cases)	
	2017–2023 [39]	EV-A	CVA6, CVA10, EV-A71	NA	NA	NA
		EV-B	CVB1–5, E5–6, E9, E11, E18	NA		
		EV-D	EV-D68	NA	Respiratory illnesses * Acute flaccid myelitis *	
European Non-Polio Enterovirus Network (ENPEN): 24 European countries	2015–2017 [11]	EV-A	CVA6 and EV-A71	19%	Neurological symptoms (45%, 3197 cases) Fever (23%, 1607 cases) Respiratory illnesses (17%, 1197 cases) Hand-foot-mouth disease (7%, 528 cases)	CSF: 35% RS: 16% Feces: 36%
		EV-B	CVB5, E5–6, E9, E11, E18, E30	46%		
		EV-D	EV-D68	5%		
Infectious Agent Surveillance Report: Japan	2014–2018 [41]	EV-A	CVA2, CVA4–6, CVA10, CVA16, EV-A71	82.8%	Hand-foot-mouth disease (58.1%, 4707 cases) Herpangina (24.4%, 1974 cases) Aseptic meningitis (17.5%, 1414 cases)	NA
		EV-B	CVB2, CVB4, E3, E6, E7, E9	7.0%		
	2019–2023 [42]	EV-A	CVA2, CVA4, CVA6, CVA10, CVA16, EV-A71	88.5%	Hand-foot-mouth disease (63.2%, 2559 cases) Herpangina (25.0%, 1011 cases) Aseptic meningitis (11.8%, 476 cases)	NA
		EV-B	CVB1, CVB5, E6, E14, E25	4.0%		
		EV-D	EV-D68	0.1%		
National Notifiable Disease Reporting and Surveillance System: China	^a^ 2013–2015 [43] HFMD surveillance	EV-A	EV-A71	37.8%	Hand-foot-mouth disease (6,604,609 cases) --With neurological or cardiopulmonary complications (0.7%, 44,742 cases)	NA
			CVA16	21.6%		
		NA	Other genotypes	40.6%		
	^b^ 2017–2019 [43] HFMD surveillance	EV-A	EV-A71	24.7%	Hand-foot-mouth disease (6,271,110 cases) --With neurological or cardiopulmonary complications (0.3%, 17,469 cases)	
			CVA16	23.7%		
		NA	Other genotypes	51.6%		
	^b^ 2017–2019 [44] HFMD surveillance	EV-A	CVA6	71.1%	Hand-foot-mouth disease (85.0%) Herpangina (10.1%)	
			CVA16	14.2%		
			EV-A71	7.0%		
			CVA2, CVA4, CVA10	4.0%		

^a^ Before and ^b^ after EV-A71 vaccine administration. * The proportion and number of cases are not available. CSF: cerebrospinal fluid; RS: respiratory sample; T/NS: throat/nasopharyngeal swab; NA: not available.

The co-circulation of EV-A, EV-B, and EV-D continued in the USA from 2017 to 2023. However, this period showed a wider range of EV genotypes, including CVA6, CVA10, EV-A71, CVB1–5, E5–6, E9, E11, E18, and EV-D68 [39]. Notably, an outbreak of EV-D68 associated with AFM reoccurred in 2018, resulting in 238 reported cases in the USA [45,46]. Although the biennial pattern of EV-D68 outbreaks and AFM cases was not observed in 2020 (during the SARS-CoV-2 pandemic), EV-D68 infections linked to respiratory illnesses were observed after the pandemic in 2022 [47]. However, the reported AFM cases in the USA remained relatively stable with 47, 34, 28, 47, and 18 cases reported in 2019, 2020, 2021, 2022, and 2023, respectively [46].

### 4.2. Enterovirus Infections in Europe

A co-circulation of EV-A, EV-B, and EV-D was observed in 24 European countries between 2015 and 2017 [11], similar to findings in the USA. During this period, ten genotypes of EV were most frequently reported, including CVA6, EV-A71, CVB5, E5–6, E9, E11, E18, E30, and EV-D68. EV-B was the most prevalent species detected and was responsible for 46% of all reported EV infections, followed by EV-A (19%) and EV-D68 (5.0%). Feces (36%) and cerebrospinal fluid (35%) were the most common specimen sources. Among EV-infected cases with recorded symptoms, neurological symptoms were the most frequent presentations (45%) that were primarily associated with EV-B infections (84% of neurological cases). Fever (23%), respiratory symptoms (17%), and HFMD (7.0%) were also reported [11]. Unlike in the USA, where EV-D68 outbreaks dominated, neurological diseases caused by EV-B were the leading cause of EV-related illnesses in Europe. The burden of HFMD caused by EV-A (7.0%) was similar to that found in the USA (6.1%). EV-D68 infections were less prevalent in Europe compared to the USA.

### 4.3. Enterovirus Infections in Japan

According to the data from Japan’s National Institute of Infectious Diseases (2014–2023), EV infections were primarily caused by EV-A [41,42]. In Japan, EV-A infections accounted for a significant majority of cases, ranging from 82.8% to 88.5% of all reported EV-infected cases. EV-B contributed to a smaller proportion of cases (4.0–7.0%), and EV-D68 was rarely detected and accounted for only 0.1% of the cases during the period of 2019–2023. Among EV-A species, the frequently reported genotypes were CVA2, CVA4–6, CVA10, CVA16, and EV-A71. Ten prevalent genotypes of EV-B were also identified (CVB1–2, CVB4–5, E3, E6, E7, E9, E14 and E25). In Japan, HFMD was the leading EV-associated illness (58.1–63.2% of the total EV-infected cases), followed by herpangina (24.4–25.0%) and aseptic meningitis (11.8–17.5%). Notably, EV-A viruses were responsible for the majority of HFMD and herpangina cases, contributing most significantly to the overall burden of EV infections. Compared to Europe, the reported rate of neurological diseases caused by EV-B in Japan appeared much lower (17.5%). However, it should be noted that Japan’s surveillance system monitors only the aseptic meningitis, excluding other neurological conditions. This limitation might lead to an underestimation-of the true burden of EV-related neurological diseases in Japan.

### 4.4. Enterovirus Infections in China

Since 2007, China’s epidemiological surveillance for EVs has focused mainly on those causing HFMD [48,49,50]. According to the National Notifiable Disease Reporting and Surveillance System, there were 15,316,710 probable HFMD cases reported between 2013 and 2019 [43]. During 2013–2015, EV-A71 and CVA16 were the predominant genotypes associated with HFMD, accounting for 37.8% and 21.6% of reported cases, respectively. Neurological or cardiopulmonary complications were observed in a relatively low number of HFMD cases (0.7%, 14,771 cases). In 2016, China introduced monovalent EV-A71 vaccines for children aged from six months to five years. Following implementation of the vaccine, the proportion of HFMD cases caused by EV-71 decreased from 38.7% to 27.7% during 2017–2019 [43]. A study in Shanghai observed a significant shift in the prevalence of EV genotypes following vaccine administration [44]. CVA6 became the most frequently detected genotype in HFMD patients from 2017 to 2019, representing 71.1% of the cases, followed by CVA16 (14.2%), EV-A71 (7.0%), CVA10 (2.6%), CVA4 (0.8%), and CVA2 (0.6%) [44]. Among all the cases collected during the epidemics of HFMD, 85.0% of the EV-infected cases were diagnosed as HFMD while 10.1% were diagnosed as herpangina. The significant impact of the EV-A71 vaccine on the epidemiology of EV-A71 was also observed in Chengdu, China. From 2017 to 2022, EV-A71-related HFMD cases accounted for only 4.2%, while cases related to CVA6, CVA16, and CVA10 accounted for 74.6%, 13.3%, and 7.9%, respectively [51]. Nevertheless, a limitation is that China’s EV surveillance system captures only HFMD, providing incomplete information regarding the overall EV infection burden in the country.

## 5. Seroepidemiology of Enteroviruses

EV seroprevalence studies can reflect the EV infections occurring in different age groups and help us to understand the susceptibility and natural infection in different populations. A large-scale longitudinal cohort study on mother–neonate pairs to assess the antibody kinetics from birth to the age of three years revealed that 66% of EV-A71 maternal antibodies were efficiently transferred to neonates. However, the antibody titer declined quickly to below a protective level at around two weeks of age [3]. Due to the rapid decline of maternal antibody titers in neonates, the proportion of neonates susceptible to EV-A71 quickly increased from 26.5% at birth to more than 90% by six months of age [52]. At 6 months to 2 years old, 85.7–93.0% of neonates were susceptible, while 63.8% and 34.4% were susceptible at 3 and 5 years old, respectively [52]. Correspondingly, after the geometric mean titer (GMT) of maternal EV-A71, antibody titers declined to the lowest point at the age of 7 months. And then, the antibody titers were gradually increased and reached the protective titer at the age of 30 months and further increased when the children grew older due to natural infection [52]. These data explained why EV infections most frequently occurred in children under 5 years old. Additionally, neonates and infants are more susceptible to viral infections of the central nervous system due to their unique anatomical and physiological characteristics, such as an incompletely developed blood-brain barrier and an immature immune system [53]. Seroepidemiological studies of antibodies against different genotypes of EV reported from several countries are summarized in Table 2.

The studies reported the seroprevalence of antibodies to different genotypes of enteroviruses, including EV-A71, CVA2, CVA4–6, CVA16, EV-D68, and E30, in the subjects with different age groups. For EV-A71, the study in China in 2010–2011 [54] showed that the seroprevalence was 28.6% at the age of 0–5 months old and decreased to 16.6%, 13.4%, and 13.9% at the age of 6–11 months, >1–2 years, and >2–3 years old, respectively. The seroprevalence increased to 24.1% and 26.1% at the age of >3–4 years and >4–5 years old, respectively. The findings indicated that the seroprevalence of 28.6% at the age of 0–5 months old was probably due to the maternal antibodies transferred from the mother to the child while the child was a fetus in the mother’s womb; it then rapidly declined to a low level after 5 months old and maintained its lowest level until 2–3 years old. The natural EV-A71 infection occurred at the age of more than 3 years old and induced the seroprevalence of the population back to a high percentage again. A similar pattern of seroprevalence dynamics of antibodies to EV-A71 was also observed in studies from Taiwan [55] and England [57]. It was unfortunate that the study from Vietnam lacked the seroprevalences at the ages of 0–5 months and 6–11 months [56]. The seroprevalence of material antibodies detected in children varied from country to country: 28.6% in China [54], 36% in Taiwan [55], and 74.3% in England [57]. In addition, the seroprevalences of children in response to natural infection of EV-A71 also differed widely from one country to another.

The seroprevalences of antibodies to CVA2, CVA4–6, and CVA16 were reported recently from China [58]. The seroprevalence dynamics pattern is more or less the same as that of EV-A71 (Table 2). The study in China [58] showed that seroprevalences to various CVA genotypes were high in children at the age of 2 months and remarkably declined to a low level at the age of 6 months and stayed at that low level until the age of 1 year. The seroprevalences of antibodies against different genotypes of CVA were gradually increased at the age of 2 years (24 months) and reached a maximum at the age of 3 years or more. In contrast, the study in England [57] revealed that the seroprevalence of antibodies against CVA6 was high (65.5%) at the age of 0–5 months and slightly decreased to 54.5% at the age of 6–11 months. The seroprevalence returned to a high percentage (64.1%) again at the age of 1–5 years and stayed high (77.8–88.1%) continuously.

The seroprevalences of antibodies against EV-D68 reported from Taiwan [59] and China [60] showed the seroprevalences in children at the age of less than 1 year and 6–11 months at 32% and 41.5%, respectively (Table 2). The study in Taiwan [59] indicated that the seroprevalence went down from 32% to 18% at the age of 1–2 years and returned back to a high level of 43% at the age of 2–3 years and reached 60% at the age of 3–5 years. The study in China [60] showed the seroprevalence of antibodies against EV-D68 at the age of 6–11 months and 1–3 years at 41.5% and 51.5%, respectively.

The study on the seroprevalence of antibodies against E30 was reported only from Korea [61], and only the seroprevalence data in children at the age of 3 months to 2 years and 3–6 years were reported at 23% and 48%, respectively (Table 2).

Overall, the seroprevalence of antibodies against various EV genotypes in children during the early life after birth (0–5 months) are at a high level, which is probably due to maternal antibodies transferred from the mother to the child while the child is a fetus in the mother’s womb; then the maternal antibodies decline rapidly to a low level at the age of 6 months to about 1–2 years. The seroprevalences return to high level again at the age of 2–3 years or more and stay at high seroprevalence because of exposure to natural infections. A similar pattern of EV seroprevalence across various age groups was consistently demonstrated among different EV genotypes.

## 6. Genotype Diversity Associated with Human Diseases

EVs cause a wide range of clinical symptoms, from mild to severe, depending on the viral genotype, the immune status of the host, and other factors. The main human diseases linked to EV infections include hand, foot, and mouth disease (HFMD), herpangina, neurological disorders, respiratory illnesses, and acute gastroenteritis (AGE). Some EV genotypes are frequently associated with multiple diseases; for example, EV-A71 has been identified as common genotype associated with HFMD, herpangina, aseptic meningitis and encephalitis, and acute flaccid myelitis, as shown in Table 3. Table 3 summarizes a comprehensive overview of various EV genotypes and their association with different human diseases.

### 6.1. Hand, Foot, and Mouth Disease (HFMD)

HFMD is a common and contagious viral illness caused by EV infections that primarily affects infants and young children. The disease usually lasts less than a week in most cases. However, a few patients may develop severe and even fatal neurological or cardiopulmonary complications [82]. Over the past few decades, there have been frequent outbreaks of HFMD worldwide, particularly in Asia-Pacific countries [95,96]. In Asia, it has been observed that over 90% of HFMD cases occur in children under 5 years old [97,98,99], with children aged 1–3 years old being the most susceptible group [52,100].

HFMD is predominantly caused by EV-A species, including EV-A71, CVA16, CVA6, CVA10, CVA2, CVA4, and CVA5 [41,42,44,62,63,64,65,101]. Among these, EV-A71 and CVA16 are the predominant pathogens causing outbreaks of HFMD worldwide [102,103,104]. In 2008, CVA6 caused an outbreak of HFMD in Finland, and later it emerged as the predominant cause of HFMD in several other European countries, the USA, and Cuba from 2009 to 2013 [105]. This trend continued in Asia, with CVA6 as the leading cause of HFMD in recent years, replacing EV-A71 and CVA16 [44,63,105,106,107]. In addition to EV-A species, EV-B species such as E3, E6, E9, E11, E15, E16, CVA9, and CVB2–5 were also reported to be associated with sporadic HFMD cases worldwide [41,66,67].

### 6.2. Herpangina

Herpangina is a common disease caused by EV infection that involves symptoms of painful sores or ulcers in the mouth and throat. Various genotypes of EVs, especially CVA, cause the disease [108]. Outbreaks of herpangina have been reported in Taiwan [68,69], Thailand [70], and mainland China [67,71]. Of note, outbreaks of herpangina and HFMD overlap fairly often, with CVA2 being more commonly identified in herpangina patients [68,69,70,71]. In addition to CVA2, other EV genotypes such as CVA16, CVA10, CVA4-5, and EV-A71 have also been associated with herpangina in several countries [41,42,72,74,75]. Less frequent genotypes like CVA9, CVB1–5, E9, and E30 have been sporadically detected in herpangina patients [1,71,73]. It is important to note that herpangina cases are not typically included in the standard EV surveillance data and therefore limit epidemiological data of the disease. The lack of data collection might lead to an underestimation of the public health burden of the diseases caused by EVs.

### 6.3. Neurological Diseases

Aseptic meningitis and encephalitis are the most common neurological complications caused by coxsackievirus B (CVB) and echovirus infections [1,34]. In the USA, EVs are responsible for a significant portion (58.4%) of aseptic meningitis and encephalitis cases in patients under 17 years old, with nearly half (45.5%) occurring in infants under one year old [109]. Notably, echovirus is frequently associated with aseptic meningitis or meningoencephalitis in neonates younger than two weeks [110]. Studies in the USA have identified echovirus strains (E3–7, 9, 11, 18, and 30), CVB1–5, and CVA9 of the EV-B species as the prevalent genotypes in patients with neurological diseases [1,39,111,112]. In Europe, 84% of EV-infected cases with neurological complications were likely associated with EV-B. Here, CVB5, E5–6, E9, E11, E18, and E30 were the most commonly detected genotypes [11]. In Japan, prevalent genotypes associated with aseptic meningitis included CVB1–5, E3, E6, E7, and E9 [41,42]. In China, EV-A71 infections can cause aseptic meningitis, encephalomyelitis, or even more severe complications like brainstem encephalitis and neurogenic pulmonary edema [76,77]. Other EV-A viruses, such as CVA2, CVA6, CVA10, and CVA16 have also been sporadically identified in aseptic meningitis or encephalomyelitis cases [78,79,113]. Acute flaccid myelitis (AFM) has been linked to various non-polio EVs. However, a definite causative association has only been confirmed for EV-A71 and EV-D68 [76,81]. Other genotypes, such as EV-D70, E33, CVA2, EV-93, and EV-94, have been detected in AFM cases, but association is much less common [80].

### 6.4. Myocarditis/Pericarditis

Coxsackievirus B (CVB1–5) is the leading cause of enteroviral myocarditis and/or pericarditis and accounts for up to 50% of the cases [34]. Approximately half of neonates (54.7%) presented clinical symptoms of myocarditis within 7 days after birth [83]. Data from the National Enterovirus Surveillance System (NESS) between 1970 and 2005 revealed that reported myocarditis cases were primarily associated with CVB1–5, with a few cases linked to CVA16 [1]. Of note, CVB1 was reported to be associated with the outbreak of myocarditis among neonates living in the Chicago area in 2007 [114]. In addition, E6, E7, and E13 genomes have also been found in the hearts of people who have died from sporadic myocarditis [84]. This suggests a link between these viruses and the disease.

### 6.5. Acute Hemorrhagic Conjunctivitis (AHC)

AHC is a highly contagious infection of the eyes characterized by a rapid onset of symptoms that typically affects both eyes. AHC is usually a mild illness that resolves on its own within a week or two. Among several EV strains, coxsackievirus A24 variant (CVA-24v) and EV-70 were identified as the common causative agent of AHC [85,86].

### 6.6. Respiratory Diseases

Between 1970 and 2005, several EV genotypes, such as CVB4, E13, and E30, were reported to be associated with respiratory diseases in the USA [1]. A large prospective epidemiological study of EV-associated respiratory infections conducted in France during 1999–2005 revealed that 31% of pediatric patients with EV infections had respiratory diseases [88]. This study showed that the EV-B species (E3, E5–7, E11, E13, E30, CVB2, and CVB4–5) were responsible for most respiratory infection cases, while the rest of cases were linked to EV-A species (EV-A71 and CVA16) [88]. However, current data suggest that EV-D68 appears to be the only human EV consistently documented as a major cause of respiratory illness [47].

### 6.7. Acute Gastroenteritis (AGE)

Although the actual causative impact of EVs on AGE has not been well-documented, several reports suggest the potential role of EVs in the disease. It has been shown that 5.1% to 11.6% and 28.8% to 51.5% of patients infected with EVs (CVA2, CVA6, CVA10, CVA16, and EV-A71) had the symptoms of diarrhea and vomiting, respectively [63,115]. This overlap in symptoms suggests that EVs potentially contribute to AGE. A case-control study found a significantly higher rate of EV infection in patients with AGE compared to those without it [116]. The EV-positive rate in patients with gastroenteritis was 2.8 times higher than those in non-gastroenteritis patients. These data further support the hypothesis of EVs playing a role in AGE. Recent studies have identified EV infections in AGE patients with the prevalences ranging from 5.3% to 26.3% of AGE patients [89,90,91,92,94,117,118]. The specific EV genotypes detected vary from study to study. In Brazil and India, E7, E9, E11, and E13 were frequently detected in patients with AGE [89,93]. E6 and E11 have been shown to associate with AGE outbreaks in Japan and India, respectively [119,120]. The CVA2, CVA4, CVA5, CVB5, CVA24, EV-C96, and PV3 (vaccine strain) have also been commonly detected in AGE patients in Japan and Thailand [90,91,92,94]. Even though these studies support the role of EVs in AGE, further studies are required to clarify the association between specific EV genotypes and AGE.

## 7. Conclusions and Future Directions

The burden of EV infections varies across different regions. In the USA, severe respiratory illness caused by EV-D68 were the primary concern from 2013 to 2023. In Europe, neurological diseases linked to EV-B infections were common in most EV-infected cases, while EV-A infections associated with HFMD were frequently reported in Asia. Currently, most countries lack standardized EV surveillance systems to monitor various diseases caused by EVs. As a result, it is challenging to accurately assess the true impact of EV infections and to provide early warnings of potential outbreaks. Therefore, establishing a standardized EV surveillance system is essential, particularly for Asian countries with frequent EV outbreaks. Furthermore, detailed clinical information and EV genotyping data from the patients must be thoroughly recorded. This information would help to identify the spectrum of the diseases associated with different EV genotypes. Further analysis of EV genotypes in combination with clinical features will establish the link between specific genotypes and related diseases. This comprehensive approach to EV surveillance will significantly improve our understanding of the global burden of EV infections and can provide an early warning of the potential outbreaks and guidelines for effective prevention and control.

## Figures and Tables

**Table 2:** Seroprevalence of antibodies to specific enterovirus genotypes in different age groups.

Genotype	Country (Study Period) [Refs.]	Seroprevalences (Age Group)						
EV-A71	China (2010–2011) [54]	28.6% (0–5 M)	16.6% (6–11 M)	13.4% (>1–2 Y)	13.9% (>2–3 Y)	24.1% (>3–4 Y)	26.1% (>4–5 Y)	
	Taiwan (1998) [55]	36% (0–5 M)	4% (6–11 M)	4% (>1–2 Y)	22% (>2–3 Y)	36% (>3–5 Y)		
	Viet Nam (2006–2007) [56]	NA	8.3% (12 M)	13.5% (18 M)	23.6% (24 M)	NA		
	England (2017) [57]	74.3% (0–5 M)	44% (6–11 M)	52.2% (1–5 Y)	69.4% (6–10 Y)	81.6% (11–20 Y)	81.3% (21–40 Y)	77.1% (>40 Y)
CVA2	China (2018) [58]	16.1% (2 M)	11.1% (6 M)	13.0% (12 M)	20.2% (24 M)	25.7% (36 M)	39.6% (48 M)	55.6% (60 M)
CVA4		66.7% (2 M)	13.8% (6 M)	13.0% (12 M)	24.5% (24 M)	39.2% (36 M)	50.0% (48 M)	68.3% (60 M)
CVA5		35.8% (2 M)	19.4% (6 M)	10.1% (12 M)	21.3% (24 M)	37.8% (36 M)	52.1% (48 M)	55.6% (60 M)
CVA6		72.8% (2 M)	25.0% (6 M)	43.5% (12 M)	47.9% (24 M)	66.2% (36 M)	81.3% (48 M)	76.2% (60 M)
CVA6	England (2017) [57]	65.5% (0–5 M)	54.5% (6–11 M)	64.1% (1–5 Y)	77.8% (6–10 Y)	88.1% (11–20 Y)	83.8% (21–40 Y)	81.6% (>40 Y)
CVA16	China (2018) [58]	45.7% (2 M)	5.6% (6 M)	5.8% (12 M)	20.2% (24 M)	32.5% (36 M)	47.9% (48 M)	49.2% (60 M)
EV-D68	Taiwan (2017) [59]	32% (<1 Y)		18% (>1–2 Y)	43% (>2–3 Y)	60% (>3–5 Y)		
	China (2012) [60]	41.5% (6–11 M)		51.5% (>1–3 Y)		NA		
E30	Korea [61]	23% (3 M–2 Y)				48% (3–6 Y)		

NA: not available; M: month; Y: year.

**Table 3:** Enterovirus genotypes associated with human diseases.

Diseases	^c^ Commonly Detected Genotypes	Uncommonly Detected Genotypes	References
Hand-foot-mouth disease	EV-A71, CVA2, CVA4–6, CVA10, CVA16	E3, E6, E9, E11, E15, E16, CVA9, CVB2–5	[41,42,44,62,63,64,65,66,67]
Herpangina	CVA2, CVA4, CVA5, CVA16, CVA10, EV-A71	CVA9, CVB1–5, E9, E25, E30	[1,41,42,68,69,70,71,72,73,74,75]
Aseptic meningitis and encephalitis	EV-A71, CVB1–5, E3–7, E9, E11, E18, E30	CVA2, CVA6, CVA10, CVA16	[1,11,41,42,76,77,78,79]
^a^ Acute flaccid myelitis	EV-A71, EV-D68	CVA7, CVA9, CVB1–6, E6–9, EV-D70 (CVA2, CVA4, CVA6, CVA14, CVA16, EV-A76, EV-119, E1–3, E11–14, E18–25, E27, E29, E30, E33, CVA20, CVA21, EV-D94)	[80,81,82]
Myocarditis/pericarditis	CVB1–5	E6, E7, E13, CVA16	[1,83,84]
Acute hemorrhagic conjunctivitis	CVA-24v, EV-A70	-	[85,86]
Respiratory diseases	EV-D68	CVA16, EV-A71, CVB2, CVB4–5, E3, E5–7, E13, E30	[1,5,87,88]
^b^ Acute gastroenteritis	CVA2, CVA4, CVA5, CVB5, E7, E9, E11, E13, CVA24, EV-C96, PV3	CVA4–6, CVA8–10, CVA16, EV-A71, CVA20–21, CVB1, E2–3, E6, E14, E18, E13, E14–15, E18, E21, E25, EV-C88, EV-C99, EV-C116	[89,90,91,92,93,94]

^a^ Genotypes with a relatively strong association with acute flaccid myelitis are shown without brackets, and those with a less common association or more recently identified ones are shown in brackets. ^b^ These data are based on the reports from Thailand, Japan, India, and Brazil. ^c^ Commonly detected genotypes refer to the enterovirus genotypes that were frequently detected in patients.

## Data Availability

Not applicable.
